# Postoperative New-Onset Heart Block in Noncardiac Surgery: Model Development, Validation, and Long-Term Prognostic Analysis

**DOI:** 10.1016/j.jacasi.2026.01.018

**Published:** 2026-03-20

**Authors:** Kai Zhang, Hao Li, Xiaoling Sha, Chang Liu, Juanjuan Gu, Qiang Fu, Yanhong Liu, Jingsheng Lou, Jiangbei Cao, Weidong Mi

**Affiliations:** a Department of Anesthesiology, The First Medical Center, Chinese People’s Liberation Army General Hospital, Beijing, China; b Medical School of Chinese People’s Liberation Army General Hospital, Beijing, China

**Keywords:** machine learning, noncardiac surgery, postoperative new-onset heart blocks, prediction model, prognosis

## Abstract

**Background:**

Heart block is common in cardiac surgery but understudied in noncardiac settings despite its poor prognosis.

**Objectives:**

This study aims to investigate epidemiology, develop predictive models, and assess the prognostic value of new-onset heart block after noncardiac surgery.

**Methods:**

This retrospective study analyzed 281,497 patients from 2008 to 2019. The primary outcome was new-onset heart block within 30 days. We used least absolute shrinkage and selection operator for variable selection and machine learning algorithms (eg, Nnet, LGBM, SVM, and XGB) for prediction. Piecewise Cox regression was used to analyze 1-year survival in elderly patients.

**Results:**

New-onset heart block occurred in 1,000 of 281,497 patients (0.36%; 95% CI: 0.33%-0.38%). Subtypes included 192 of 281,497 atrioventricular (AV) blocks (0.07%; 95% CI: 0.06%-0.08%); 51 of 281,497 left bundle branch blocks (0.02%; 95% CI: 0.01%-0.02%); and 757 of 281,497 right bundle branch blocks (RBBBs) (0.27%; 95% CI: 0.25%-0.29%). The XGB model achieved a validation area under the curve of 0.804 using 12 predictors. Median follow-up for elderly patients was 162 days (Q1-Q3: 85-256) for nonsurvivors and 365 days (Q1-Q3: 365-365) for survivors. AV block predicted poor early-phase prognosis (adjusted HR: 6.24; 95% CI: 1.83-21.23), whereas RBBB was associated with poor late-phase prognosis (adjusted HR: 2.67; 95% CI: 1.26-5.69).

**Conclusions:**

New-onset heart block affects 0.36% of noncardiac surgery patients. Machine learning effectively predicts this complication. Given the significant mortality risks associated with AV block and RBBB, enhanced perioperative surveillance is crucial.

With the continuous advancement of medical technology, surgery is gradually becoming the mainstay of disease treatment, which puts forward higher requirements for the perioperative management of patients. The goals of postoperative management include reducing pain, accelerating postoperative recovery, and minimizing the occurrence of complications. At present, the focus of postoperative complications is mostly on the prevention and treatment of serious complications, such as myocardial infarction, delirium, stroke, and death^1^, ^2^, ^3^; less attention is paid to some asymptomatic, mild complications. Heart blocks are common, and most are clinically benign (except for second- and third-degree atrioventricular [AV] blocks). The incidence of heart block after cardiac surgery ranges from 5.8% to 38.3%.^4^, ^5^, ^6^, ^7^, ^8^, ^9^ In recent years, several studies have found that the development of heart block after cardiac surgery is associated with a poor long-term prognosis. For example, in a multicenter study of left bundle branch block (LBBB) after transcatheter aortic valve implantation, patients with new-onset LBBB after surgery had a 1.54-fold higher risk of death.^10^

Heart blocks also occur after noncardiac surgery; however, few studies have focused on their complications. In one case report, a patient developed sudden cardiac discomfort after open partial nephrectomy and was diagnosed with LBBB using electrocardiography (ECG).^11^ However, the authors were unclear about the cause of LBBB and could only speculate and treat the complications based on previous experience; none of the treatments worked. Therefore, understanding the prognostic value of these complications in noncardiac surgery and exploring their risk factors is helpful for prevention and management. Traditional statistical methods have been widely used in previous studies to explore risk factors and establish predictive models. In recent years, machine-learning (ML) techniques have been widely used in big data research.^12^, ^13^, ^14^ This technique can understand the complex relationships between variables in a flexible and trainable way, and the Shapley additive explanations (SHAP) method can interpret this complex relationship.^15^

Therefore, in this retrospective cohort study, we used information from the patients’ electronic medical record to investigate the epidemiology of new-onset heart block after noncardiac surgery, established and validated prediction models based on traditional statistical methods and ML algorithms, and explored the prognostic value of new-onset heart block after noncardiac surgery.

## Methods

### Setting and data sources

Data were collected from the electronic medical records of all patients who underwent noncardiac surgery at the Chinese PLA General Hospital between January 1, 2008, and August 1, 2019. Patients were excluded if the surgical duration was <30 minutes or if they were younger than 18 years of age. Data elements were extracted from patient anesthesia and medical records and used to construct and validate the prediction model in this study.

This study was approved by the Research Ethics Committee of the First Medical Center of the Chinese PLA General Hospital (Approval Reference No. S2023-438-01). This study was registered at ClinicalTrials.gov (NCT06119009). The study adhered to the TRIPOD (Transparent Reporting of a Multivariable Prediction Model for Individual Prognosis or Diagnosis) criteria.^16^

### Outcomes

The primary outcome was postoperative new-onset heart block, which was defined as new-onset heart block within 30 days after surgery, including first-, second-, and third-degree AV blocks and left- and right-branch bundle blocks (RBBB). If patients experienced the same type of heart block that occurred within 3 months before the surgery, the heart block was not defined as new onset. Study outcomes were confirmed using 12-lead ECGs and professional ECG room physicians.

### Data collection

Based on clinical experience and previous relevant studies**,**^17^^,^^18^ we identified variables potentially associated with the occurrence of new-onset postoperative cardiac conduction block, including the following: 1) demographic details such as age, sex, and body mass index (BMI); 2) medical history covering cerebrovascular disease, coronary heart disease (CHD), valvular heart disease, heart failure, arrhythmia, hypertension, diabetes mellitus, cardiac interventions (eg, coronary artery bypass grafting, percutaneous coronary intervention), and peripheral arterial disease; 3) preoperative medications, including angiotensin-converting enzyme inhibitors, angiotensin receptor blockers, β-blockers, calcium channel blockers, antiplatelet agents, and anticoagulants; 4) the latest presurgery laboratory results, including white blood cell (WBC) and red blood cell (RBC) counts, hemoglobin, serum creatinine (SCr), blood glucose, fibrinogen (FB), serum albumin, serum potassium, and blood calcium levels; and 5) surgery-related factors, including the American Society of Anesthesiologists **(**ASA**)** score, type of surgery and anesthesia, surgery duration, emergency surgery status, blood loss, crystal infusion rate, and duration of intraoperative hypotension. Intraoperative hypotension was characterized by a mean arterial pressure <65 mm Hg.^19^

The prognosis outcome was overall survival for elderly patients (≥65 years of age), defined as the time between the date of surgery and the day of death following surgery for 1 year.^20^ One-year postoperative survival data were collected from the patients’ medical records and verified by telephone follow-up at the Chinese Center for Disease Control and Prevention.

### Sample size estimation

An a priori sample size calculation was not performed because of the lack of comparable prior studies and the primary objective of developing a predictive model. However, to ensure sufficient statistical power and robustness of the multivariable analysis, we adhered to the **“**events**-**per**-**variable**”** principle. Specifically, to prevent overfitting, the number of covariates included in the model was restricted to less than one-tenth of the total number of outcome events.

### Statistical analysis

Continuous variables with a normal distribution are represented as the mean ± SD, whereas those with a non-normal distribution are represented as median (Q1-Q3). Categorical data are presented as frequencies (%). Continuous variables were compared using the Mann–Whitney U test or independent samples Student’s t-test, whereas categorical variables were assessed using the chi square test or Fisher exact test, depending on suitability.

First, we established prediction models for postoperative new-onset heart block using traditional statistical analysis methods and ML algorithms. The dataset was randomly divided into a training set (80%) and an internal validation set (20%). Logistic regression analysis was used to develop a traditional prediction model visualized using a nomogram. The discrimination of the model was assessed using the area under the receiver operating characteristic curve (AUC) in both the training and validation sets, and its calibration was evaluated using a calibration curve. ML prediction models were developed using 4 distinct algorithms: neural network classifier (Nnet), light gradient boosting machine (LGBM), support vector machine (SVM), and extreme gradient boosting (XGB) with classification trees. We used 10-fold cross-validation on the training set to create an unbiased prediction model. AUC was used to discriminate the prediction model in the training and internal validation sets. The SHAP method was used to interpret the ML prediction models.

Least absolute shrinkage and selection operator logistic regression analysis was initially used to select predictors from the collected variables. Subsequently, univariate and multivariate logistic regression analyses were performed for the remaining variables. Variables with clinical significance (*P* < 0.1) in the univariate analysis were included in the backward stepwise multivariate logistic regression. Subsequently, 16 variables are selected. To simplify the prediction model indicators, we reduced the number of model indicators to 12 to ensure the efficiency of the prediction models. These 12 variables contributed the most to the AUC in the logistic regression prediction model and were relatively high in the importance ranking of the 4 ML prediction models.

We explored the relationship between different types of postoperative new-onset heart blocks and postoperative 1-year overall survival in elderly patients. The proportional hazards (PH) assumption was systematically verified using scaled Schoenfeld residuals. The results indicated that the PH assumption was valid for the primary exposure variable (heart block types, *P =* 0.177) and covariates, including sex, cerebrovascular disease, diabetes, and arrhythmia. However, this assumption was violated for age (*P* < 0.001), ASA score (*P =* 0.004), and surgery type (*P* < 0.001), resulting in a significant global test result (*P* < 0.001). To account for the non-PH of these covariates, a piecewise Cox regression model was used. The follow-up period was stratified into 4 intervals: early (0-3 months), early-middle (3-6 months), late-middle (6-9 months), and late (9-12 months). The selected confounding variables included age, sex, history of cerebrovascular disease, diabetes, arrhythmia, ASA score, and surgery type, which showed significant differences (*P* < 0.01) between the groups of survivors and those who died 1 year after surgery and are acknowledged to influence the quality of life of patients.

Missing values were handled appropriately to maximize data use. Variables with more than 20% missing data were excluded from the analysis. For variables with a missing rate of less than 20% (eg, BMI or laboratory indices), missing values were imputed using the median (for continuous variables with a skewed distribution) or mean (for normally distributed variables). Categorical variables were analyzed using this mode.

The significance level was set at a 2-sided *P* < 0.05. Statistical analyses in this study were performed using R version 3.6.3 and Python programming language version 3.6.

## Results

**Figure 1:**
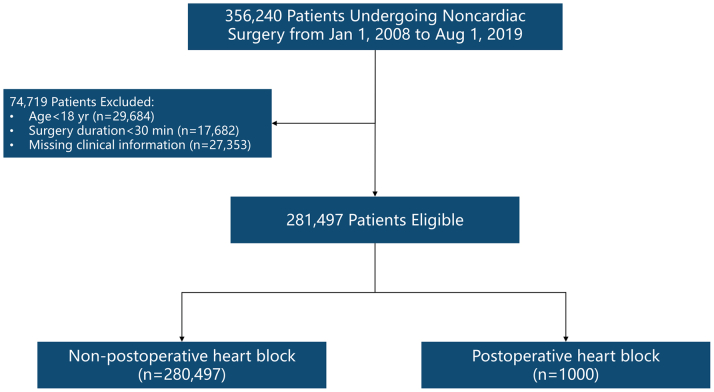
Patient Selection and Study Design Flowchart The inclusion and exclusion criteria applied to the initial cohort of patients who underwent noncardiac surgery. Patients with preexisting cardiac conduction disorders or incomplete data were excluded from the database to ensure data quality. The final study population was randomly divided into training and validation sets for the development and testing of machine learning models. This rigorous screening process defined the denominator for calculating the 0.36% incidence rate.

From an initial cohort of 356,240 patients undergoing noncardiac surgery, 74,743 were excluded. As shown in Figure 1, data from 281,497 patients were analyzed. A total of 1,000 patients had postoperative heart blocks (n = 1,000 of 281,497; 0.36%). Among these, AV blocks occurred in 192 patients (n = 192 of 281,497; 0.07%; 95% CI: 0.06%-0.08%), LBBB in 51 (n = 51 of 281,497; 0.02%; 95% CI: 0.01%-0.02%), and RBBB in 757 (n = 757 of 281,497; 0.27%; 95% CI: 0.25%-0.29%), as shown in Table 1.

**Table 1:** Incidence and Classification of Postoperative New-Onset Heart Blocks (N = 281,497)

Outcomes	Incidence
Total heart blocks	1,000 (0.36); 0.33-0.38
AV blocks	192 (0.07); 0.06-0.08
First-degree AV block	172 (0.06); 0.05-0.07
Type-Ⅰ second-degree AV block	18 (0.01); 0-0.01
Type-II second-degree AV block	1 (0)
Third-degree AV block	1 (0)
LBBB	51 (0.02); 0.01-0.02
RBBB	757 (0.27); 0.25-0.29

Values are n (%); 95% CI.

AV = atrioventricular block; LBBB = left bundle branch block; RBBB = right bundle branch block.

### Clinical characteristics of the baseline data

Detailed clinical characteristics of the patients are shown in Table 2. Compared with patients without postoperative heart blocks, the patients with postoperative heart blocks were older (median age, 62 [Q1-Q3: 52**-**72] years vs 51 [Q1-Q3: 39**-**61] years**;** *P* < 0.001), more female (n = 622 of 1,000 [62.2%] vs n = 133,870 of 280,497 [47.7%]; *P* < 0.001), and more with history of diseases including cerebrovascular disease, CHD, valvular heart disease, heart failure, arrhythmia, HT, diabetes mellitus, conditions requiring cardiac interventions, and peripheral arterial disease. Corresponding to the higher proportion of patients with a history of cardiovascular disease in patients with new-onset heart block after surgery, these patients also had a higher proportion of preoperative medication for cardiovascular diseases. For preoperative laboratory test results, there were also differences between the two groups of patients. For example, patients with new-onset heart block after surgery had lower preoperative hemoglobin and blood calcium levels but higher SCr, WBC, and FB levels. For surgery-related variables, there were significant differences in the surgical specialties between the two groups. Besides, the patients with postoperative heart blocks had longer surgery duration (>3 hours: n = 436 of 1,000 [43.6%] vs n = 78,690 of 280,497 [28.1%]; *P* < 0.001), higher ASA scores (ASA >Ⅱ: n = 352 of 1,000 [35.2%] vs n = 22,715 of 280,497 [8.1%]; *P* < 0.001) and more proportion of intravenous anesthesia combined with inhalational anesthesia (n = 796 of 1,000 [79.6%] vs n = 205,027 of 280,497 [73.1%]; *P* < 0.001), more proportion of emergency surgery (n = 154 of 1,000 [15.4%] vs n = 14,002 of 280,497 [4.99%]; *P* < 0.001), more blood loss [intraoperative blood loss exceeding 300 mL; blood loss n = 494 of 1,000 [49.4%] vs n = 89,770 of 280,497 [32.0%]; *P* < 0.001), and longer duration of intraoperative hypotension (5 [Q1-A3: 0-15] min vs 0 [Q1-Q3: 0-10] min**;** *P* < 0.001).

**Table 2:** Baseline Characteristics Stratified by New-Onset Heart Block Status

	Non-Heart Block (n = 280,497)	Heart Block (n = 1,000)	*P* Value
Demographic characteristics			
Male	146,627 (52.3)	378 (37.8)	<0.001
Age, y	51 (39-61)	62 (52-72)	<0.001
BMI, kg/m^2^	24.3 (22.0-26.9)	24.2 (21.9-26.8)	0.207
Previous history			
Cerebrovascular diseases	12,689 (4.52)	92 (9.20)	<0.001
Arrhythmia	9,447 (3.37)	123 (12.3)	<0.001
Peripheral artery disease	4,620 (1.7)	58 (2.6)	0.001
Heart failure	330 (0.1)	41 (1.8)	<0.001
Hypertension	60,735 (21.7)	372 (37.2)	<0 0.001
Diabetes mellitus	36,935 (13.2)	199 (19.9)	<0.001
Coronary heart disease	22,998 (8.20)	220 (22.0)	<0.001
Valvular heart disease	800 (0.29)	10 (1.00)	0.001
Conditions requiring cardiac interventions	2,098 (0.75)	41 (4.10)	<0.001
Preoperative medication			
ACEI	5,417 (1.93)	31 (3.10)	0.01
ARB	10,996 (3.92)	67 (6.70)	<0.001
β-blockers	10,957 (3.91)	123 (12.3)	<0.001
Calcium channel blockers	32,847 (11.7)	201 (20.1)	<0.001
Antiplatelet	7,508 (2.68)	84 (8.40)	<0.001
Anticoagulation drugs use	14,883 (5.31)	158 (15.8)	<0.001
Preoperative laboratory data			
High RBC	12,772 (4.55)	158 (15.8)	<0.001
High WBC	17,390 (6.20)	170 (17.0)	<0.001
Hb, g/dL	133 (122-146)	129 (112-142)	<0.001
SCr, mol/L	0.74 (0.63-0.87)	0.78 (0.66-0.95)	<0.001
High blood glucose	39,074 (13.9)	317 (31.7)	<0.001
FB, g/L	3.05 (2.59-3.70)	3.42 (2.81-4.25)	<0.001
High serum albumin	2,686 (0.96)	12 (1.20)	0.533
Serum potassium, mEq/L	3.98 (3.78-4.21)	4.01 (3.77-4.25)	0.091
Blood calcium, mg/dL	2.25 (2.19-2.32)	2.23 (2.14-2.30)	<0.001
Operation-related factors			
Surgery duration >3 hours	78,690 (28.1)	436 (43.6)	<0.001
ASA score >II	22,715 (8.10)	352 (35.2)	<0.001
Emergency surgery	14,002 (4.99)	154 (15.4)	<0.001
Crystalloid infusion rate, mL/kg/min	0.15 (0.11-0.21)	0.15 (0.11-0.20)	0.036
High blood loss	89,770 (32.0)	494 (49.4)	<0.001
Surgical type			<0.001
Reproductive	31,422 (11.2)	32 (3.20)	
Orthopedics	57,375 (20.5)	192 (19.2)	
Urology	21,137 (7.54)	56 (5.60)	
General	39,936 (14.2)	310 (31.0)	
Neurosurgery	21,596 (7.70)	43 (4.30)	
Thoracic	19,989 (7.13)	133 (13.3)	
Vascular	6,485 (2.31)	58 (5.80)	
Others	45,964 (16.4)	62 (6.20)	
Hepatobiliary	36,593 (13.0)	114 (11.4)	
Anesthesia type			<0.001
Intravenous anesthesia combined with local anesthesia	13,254 (4.73)	40 (4.00)	
Intravenous anesthesia combined with inhalational anesthesia	205,027 (73.1)	796 (79.6)	
Intravenous anesthesia	40,065 (14.3)	131 (13.1)	
Local anesthesia	17,065 (6.08)	0 (0.00)	
Intravenous anesthesia combined with inhalational anesthesia+local anesthesia	5,086 (1.81)	33 (3.30)	
Duration of intraoperative hypotension, min	0 (0-1)	0.5 (0-1.5)	<0.001

Values are n (%) or median (Q1-Q3).

ACEI = angiotensin-converting enzyme inhibitors; ARB = angiotensin receptor blocker; ASA = American Society of Anesthesiologists; BMI = body mass index; FB = fibrinogen; Hb = hemoglobin; RBC = red blood cell; SCr = serum creatinine; WBC = white blood cell.

### Establishment of prediction models

**Figure 2:**
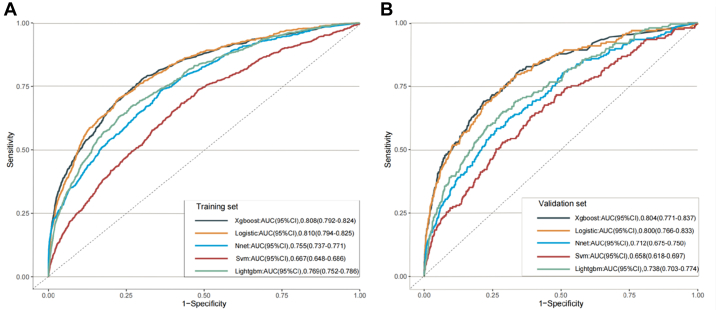
Discriminative Performance of Machine Learning Models Receiver operating characteristic curves comparing the predictive accuracy of the 5 different algorithms in the (A) training set and (B) validation set. The extreme gradient boosting (XGB) model achieved an area under the curve (AUC) of 0.808 in the training set and 0.804 in the validation set, demonstrating robust performance comparable to that of logistic regression. The proximity of the curves suggests that the selected variables are strong predictors regardless of the algorithm used, thus validating the clinical utility of the final model. Nnet = neural network classifier; SVM = support vector machine.

**Figure 3:**
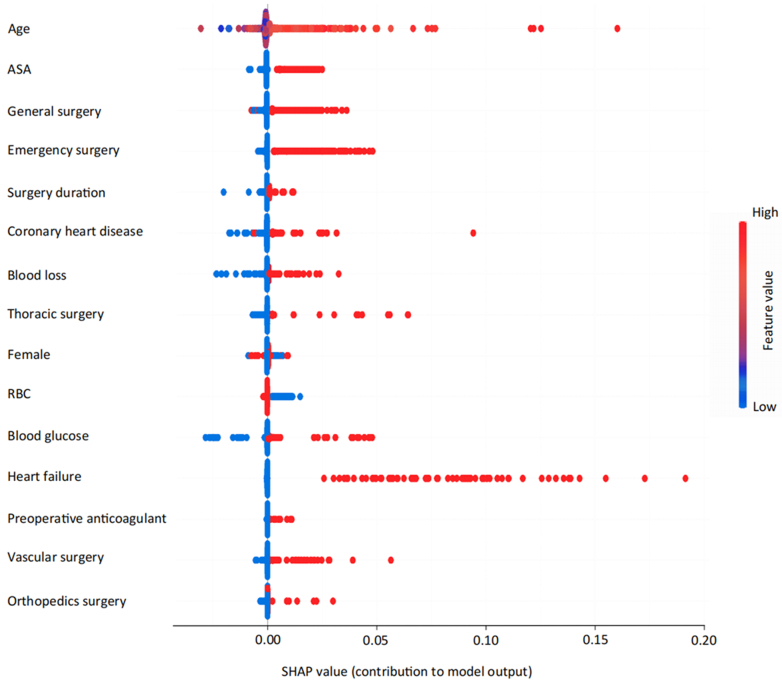
Feature Importance Visualization in the XGB Prediction Model The Shapley Additive Explanations (SHAP) summary plot shows the contribution of the top predictive features to the risk of developing postoperative new-onset heart block. The features were sorted by importance, with age and American Society of Anesthesiologists (ASA) scores showing the strongest predictive values. Each dot represents a patient; red indicates a high feature value (eg, older age), and blue indicates a low value. The horizontal location shows whether the feature increases (right) or decreases (left) risk. This model provides interpretability for clinical decision-making. RBC = red blood cell.

Using least absolute shrinkage and selection operator logistic regression analysis, 30 variables were retained after excluding preoperative FB variables (Supplemental Figures 1A and 1B). The remaining variables were then used in the univariate and multivariate logistic regression analyses of the training set. After multivariable logistic regression analysis, 16 variables remained (Table 3): age, female sex, history of arrhythmia, history of CHD, history of heart failure, preoperative anticoagulant use, preoperative β-blockers use, preoperative abnormal WBC, preoperative RBC, preoperative high glucose, preoperative blood calcium, ASA score >II, surgical specialty, surgery duration >3 hours, emergency surgery**,** and blood loss. Next, we reduced the number of indicators to 12 for the simplification of the prediction models: age, female sex, history of CHD, history of heart failure, ASA score >II, surgical specialty, emergency surgery, blood loss, preoperative anticoagulant use, preoperative RBC, preoperative high glucose, and surgery duration >3 hours (Supplemental Figure 2). The AUCs of the training set and validation set remained relatively stable, with AUCs of 0.810 (95% CI: 0.794-0.825) and 0.800 (95% CI: 0.766**-**0.833) in the training set and validation set, respectively (Figures 2A and 2B). We then used the same predictive indicators and training and validation sets to establish prediction models using 4 ML algorithms: Nnet, LGBM, SVM, and XGB. The AUCs of these models for the training and validation sets are shown in Figures 2A and 2B, respectively. The hyperparameters of each prediction model are listed in (Supplemental Table 1). As shown in the figures, the prediction model established that using XGB had a higher AUC. The AUCs of the XGB model in the training and validation sets were 0.808 (95% CI: 0.792-0.824) and 0.804 (95% CI: 0.771-0.837), respectively, which were comparable to the AUCs of the prediction model established using the logistic regression model. As shown in Figure 3, we used SHAP values to explain the predictive indicators in the XGB machine learning prediction model. As shown in Figure 3, age, female sex, history of CHD, history of heart failure, ASA score >II, surgical specialty, emergency surgery, blood loss, preoperative anticoagulant use, preoperative hyperglycemia, and surgery duration >3 hours might be risk factors for developing heart block after noncardiac surgery, whereas a higher preoperative RBC count might be a protective factor. In terms of surgical type, heart block was more likely to occur after general surgery (70% were gastrointestinal surgeries) than after thoracic, vascular, or orthopedic surgery.

**Table 3:** Univariate and Multivariable Analyses of Predictors for New-Onset Heart Blocks

	Univariate Analysis	Multivariable Analysis	*P* Value
Age	1.05 (1.05-1.06)	1.03 (1.02-1.03)	<0.001
Female	1.88 (1.63-2.17)	1.41 (1.19-1.66)	<0.001
BMI	0.99 (0.97-1.01)		0.163
History of cerebrovascular diseases	2.17 (1.71-2.76)		<0.001
History of arrhythmia	4.15 (3.37-5.12)	1.71 (1.36-2.16)	<0.001
History of peripheral artery disease	1.98 (1.34-2.93)		<0.001
History of heart failure	29.66 (19.98-44.04)	4.95 (3.14-7.80)	<0.001
History of hypertension	2.14 (1.86-2.47)		<0.001
History of diabetes mellitus	1.62 (1.36-1.93)		<0.001
History of coronary heart disease	3.11 (2.63-3.68)	1.75 (1.43-2.15)	<0.001
History of valvular heart disease	3.50 (1.73-7.05)		<0.001
History of conditions requiring cardiac interventions	5.64 (3.97-8.02)		<0.001
ACEI	1.69 (1.14-2.50)		0.009
ARB	1.78 (1.35-2.35)		<0.001
β-Blockers	3.36 (2.71-4.15)	1.29 (1.01-1.66)	0.042
Calcium channel blockers	1.84 (1.55-2.20)		<0.001
Antiplatelet	3.41 (2.66-4.37)		<0.001
Anticoagulation drugs use	3.12 (2.56-3.79)	1.29 (1.01-1.64)	0.042
High RBC	0.26 (0.21-0.31)	0.67 (0.51-0.87)	0.003
High WBC	3.08 (2.56-3.71)	1.64 (1.30-2.06)	<0.001
Hb	0.98 (0.98-0.99)		<0.001
SCr	1.09 (1.05-1.13)		<0.001
High blood glucose	2.75 (2.36-3.19)	1.34 (1.12-1.60)	0.001
FB	1.32 (1.26-1.39)		<0.001
High serum albumin	1.31 (0.70-2.44)		0.403
Serum potassium	1.15 (0.95-1.40)		0.154
Blood calcium	0.04 (0.03-0.07)	0.53 (0.32-0.89)	0.016
Surgery duration >3 hours	2.08 (1.81-2.39)	1.31 (1.10-1.55)	0.002
ASA score >II	6.10 (5.27-7.06)	2.00 (1.67-2.39)	<0.001
Emergency surgery	3.37 (2.78-4.09)	2.84 (2.20-3.67)	<0.001
Crystalloid infusion rate	0.64 (0.30-1.38)		0.259
High blood loss	2.16 (1.88-2.48)	1.66 (1.40-1.96)	<0.001
Orthopedics surgery	3.38 (2.21-5.15)	1.90 (1.21-2.99)	0.005
Urology surgery	2.60 (1.59-4.26)	1.86 (1.10-3.15)	0.021
General surgery	8.02 (5.32-12.09)	4.85 (3.11-7.58)	<0.001
Neurosurgery surgery	1.79 (1.06-3.04)		0.03
Thoracic surgery	6.69 (4.33-10.34)	4.71 (2.97-7.56)	<0.001
Vascular surgery	8.55 (5.23-13.97)	3.72 (2.17-6.37)	<0.001
Others surgery	1.39 (0.86-2.25)		0.176
Hepatobiliary surgery	3.08 (1.97-4.79)		<0.001
Intravenous anesthesia combined with inhalational anesthesia	1.27 (0.89-1.80)		0.187
Intravenous anesthesia	0.98 (0.66-1.45)		0.919
Intravenous anesthesia combined with inhalational anesthesia+local anesthesia	1.98 (1.18-3.34)		0.010
Duration of intraoperative hypotension	1.05 (1.03-1.07)		<0.001

Values are OR (95% CI).

OR = odds ratio; other abbreviations as in Table 2.

### Postoperative new-onset heart blocks and long-term prognosis in elderly patients

**Figure 4:**
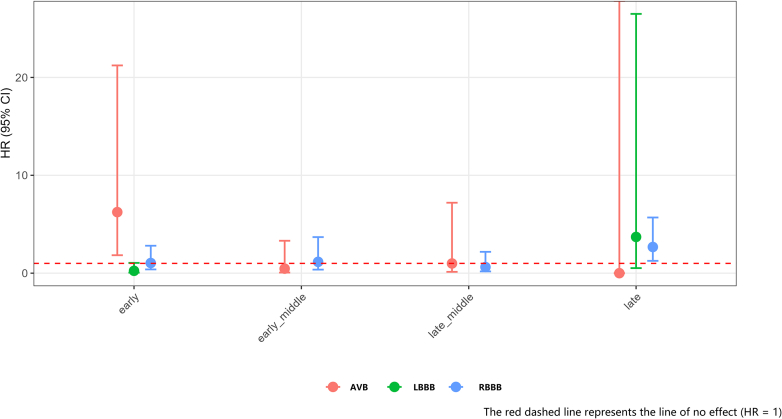
New-Onset Heart Block and Prognosis in Elderly Noncardiac Surgery Piecewise Cox regression analysis showed an association between specific types of new-onset heart block and 1-year all-cause mortality in an elderly cohort. The graph highlights the time-varying effects: new-onset atrioventricular block (AVB) (red line) is significantly associated with an HR of 6.24 in the early postoperative phase, indicating immediate risk. In contrast, new-onset right bundle branch block (RBBB) (blue line) was associated with an HR of 2.67 in the late phase. The shaded areas represent 95% CIs.

A total of 50,286 elderly patients were included in this study (49,866 non-postoperative heart block patients and 420 postoperative heart block patients). A total of 1,532 patients died within 1 year after surgery. The median follow-up time was 162 (Q1-Q3: 85-256) days for nonsurvivors and 365 (Q1-Q3: 365-365) days for survivors. Among patients with postoperative heart blocks, 25 (5.9%) died within 1 year after surgery. Among the 49,866 non-postoperative heart block patients, 1,507 (3.0%) died within 1 year of surgery. Within 30 days after surgery, new-onset AV block, LBBB, and RBBB were observed in 92, 30, and 285 elderly patients, respectively. As shown in Figure 4, the piecewise Cox regression model demonstrated time-varying effects: new-onset AV block was significantly correlated with a poor 1-year prognosis in the early phase (adjusted HR [aHR]: 6.24; 95% CI: 1.83**-**21.23; *P* < 0.01), whereas new-onset RBBB was significantly associated with poor prognosis in the late phase (aHR: 2.67; 95% CI: 1.26-5.69; *P* < 0.05).

## Discussion

As summarized in the Central Illustration, we conducted a retrospective study including 281,497 noncardiac surgery patients with 1**,**000 patients who developed postoperative new-onset heart blocks (first-, second-, and third-degree AV blocks and LBBB and RBBB) and found an association between postoperative new-onset AV blocks and RBBB with long-term mortality in elderly patients. In addition, we established prediction models using a traditional logistic regression method and 4 ML algorithms. Among the prediction models constructed using ML algorithms, the model constructed using the XGB algorithm had the highest predictive ability, with AUCs of 0.808 (95% CI: 0.792**-**0.824) and 0.804 (95% CI: 0.771**-**0.837) for the training and validation sets, respectively. These AUCs were comparable to the AUCs of the prediction model constructed using the logistic regression model for the training and validation sets (0.810; 95% CI: 0.794**-**0.825 and 0.800; 95% CI: 0.766**-**0.833), respectively.

**Central Illustration:**
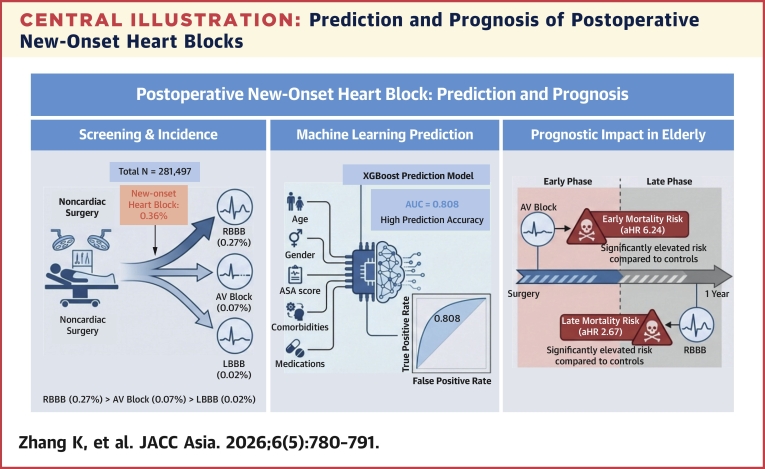
Prediction and Prognosis of Postoperative New-Onset Heart Blocks In this study, we analyzed 281,497 patients who underwent noncardiac surgery to investigate the incidence and prognosis of new-onset heart block. The incidence was 0.36%, with right bundle branch block (RBBB) being the most common. An extreme gradient boosting (XGB) machine-learning model using 12 key variables achieved an area under the curve (AUC) of 0.808 for risk prediction. Survival analysis in elderly patients revealed a time-dependent risk; atrioventricular (AV) block was associated with high mortality in the early postoperative phase, whereas RBBB was correlated with poor prognosis in the late phase, highlighting the need for prolonged monitoring. ASA = American Society of Anesthesiologists; LBBB = left bundle branch block.

Our results indicated that the incidence of new-onset heart block after noncardiac surgery was 0.36%, which was significantly higher than that observed in the general population.^21^^,^^22^ Notably, within the optimal prediction model constructed in this study, surgery-related variables, including ASA score, type of surgery, and intraoperative blood loss, demonstrated a high predictive value. Collectively, these findings suggest that noncardiac surgery itself may increase the risk of postoperative new-onset heart block. However, whereas the existing literature has predominantly focused on the prevention and treatment strategies for heart block following cardiac surgery, there is a marked paucity of attention regarding this complication in the context of noncardiac surgery. By analyzing the epidemiological characteristics of new-onset heart block after noncardiac surgery, this study revealed that the actual incidence of this complication may exceed previous expectations and has significant clinical implications. Therefore, the established risk prediction model provides an effective tool for the early clinical identification of high-risk patients, contributes to the optimization of perioperative management strategies, and provides evidence for advancing the construction of a precise perioperative cardiac monitoring system.

By integrating our SHAP results with those of previous studies, we propose that postoperative heart block arises from the interaction between multiple perioperative insults and an intrinsically susceptible substrate. First, the high predictive contribution of age, CHD, and heart failure highlights a compromised anatomical basis. Age-related degenerative fibrosis, superimposed on chronic ischemia and myocardial remodeling, weakens the conduction system’s physiological reserve, creating a “susceptible state.”^23^^,^^24^ Second, the duration of surgery, blood loss, and RBC count were significant indicators of oxygen supply and demand imbalance. Reduced oxygen-carrying capacity due to anemia, combined with perfusion fluctuations during prolonged surgery, may cause acute ischemic injury in hypoxia-sensitive conduction tissues.^25^^,^^26^ Finally, the impact of emergency/thoracic surgery and hyperglycemia implicate neural regulation and inflammatory mechanisms. Surgical stress and hyperglycemia-induced systemic inflammatory response syndrome can impair ion channels. Additionally, severe autonomic imbalance or vagal stimulation may act as triggers for heart blocks in high-risk patients.^27^, ^28^, ^29^, ^30^, ^31^, ^32^, ^33^, ^34^

Beyond identifying noncardiac surgery as a risk factor for heart block, this study highlights the significant prognostic impact of postoperative heart block on long-term outcomes. Using piecewise Cox regression, we revealed a clinically important finding, namely**,** that different types of new-onset postoperative heart block exert time-dependent effects on patient prognosis. Specifically, new-onset AV block primarily increases early postoperative mortality, whereas new-onset RBBB significantly worsens late outcomes. This “early-late” difference likely reflects distinct pathophysiological consequences. The emergence of AV block may serve as an early sensitive marker of acute myocardial ischemia or inflammatory injury to the AV nodes. In the perioperative setting with an elevated sympathetic tone, delayed conduction suggests acute decompensation of the conduction system, which correlates strongly with myocardial injury, thereby increasing the risk of fatal early cardiovascular events.^35^^,^^36^ In contrast, the adverse late impact of new-onset RBBB reflects the cumulative burden of the underlying cardiovascular disease and ventricular remodeling. Previous studies have linked RBBB to extensive coronary artery disease, right ventricular pressure overload (eg, pulmonary pathology), and myocardial fibrosis.^37^^,^^38^ The abnormal ventricular activation sequence induced by RBBB causes ventricular asynchrony, which progressively impairs cardiac pump efficiency and precipitates or exacerbates heart failure over time.^39^^,^^40^ Thus, new-onset RBBB may act as a red flag, signaling severe baseline cardiovascular pathology or ongoing adverse ventricular remodeling, a chronic process that accumulates over the postoperative year and ultimately manifests as increased late mortality.

### Study limitations

First, the incidence of postoperative new-onset heart blocks was relatively lower than the actual incidence rate because this was a retrospective cohort study, and data were obtained from medical records with inevitably missing follow-up data. Second, the information was gathered from a single hospital; however, as a large tertiary hospital, the patients came from all over the country, and the reliability of the study findings was increased by collecting data over 12 years, including 281,497 patients. Additionally, although internal cross-validation was used for the prediction model in this study, external validation of the model is required. Third, we were unable to collect variable information, such as echocardiographic data and detailed patient information, 1 month after surgery. In the future, we plan to conduct prospective research to further validate the relationship between the occurrence of postoperative new-onset heart block and the prognosis of noncardiac surgery patients.

## Conclusions

In this study, the incidence of new-onset heart block within 30 days after noncardiac surgery was 0.36%. The prediction models developed in this study effectively identified patients at risk of this complication. Furthermore, the significant association between new-onset hepatitis blocks and long-term mortality in elderly patients underscores the critical importance of enhancing perioperative management to improve outcomes.

## Funding Support and Author Disclosures

This research was supported by the National Key Research and Development Program of China (2018YFC2001900) and the Beijing Nova Program (Z211100002121171). The authors have reported that they have no relationships relevant to the contents of this paper to disclose.
