# Supplementary material for: Postoperative New-Onset Heart Block in Noncardiac Surgery: Model Development, Validation, and Long-Term Prognostic Analysis
Source: JACC Asia. 2026 Mar 20;6(5):780–91. doi: 10.1016/j.jacasi.2026.01.018 (PMC13153885; doi:10.1016/j.jacasi.2026.01.018)
Supplement: Supplemental Figures 1 and 2 and Supplemental Table 1 [file mmc1.docx]

**Supplemental Figure 1**. Variable Selection Using LASSO Regression Analysis


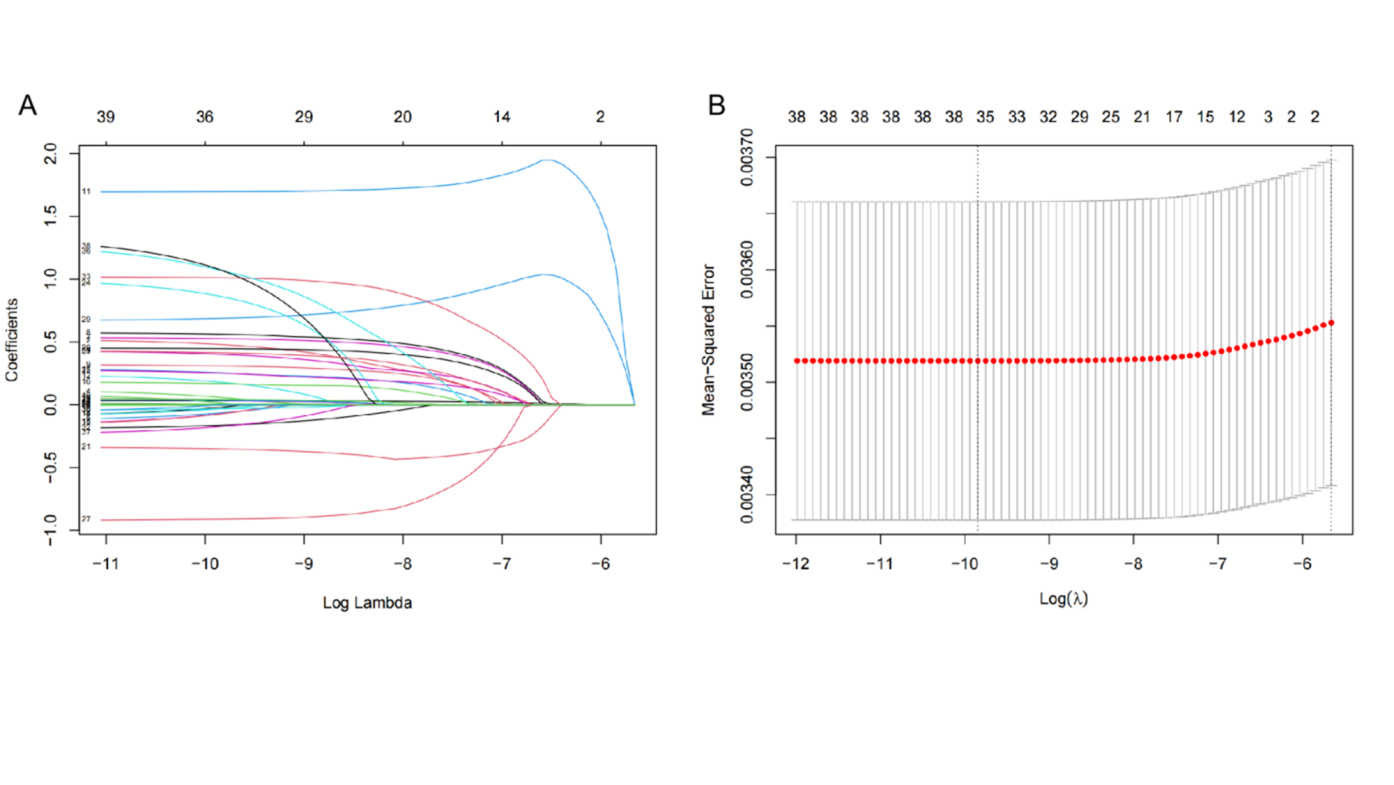


(A) LASSO coefficient profiles of 30 candidate predictors. Each line represents a variable, and as the penalty parameter (lambda) increases, the coefficients shrink toward zero. (B) The partial likelihood deviance plotted against log(lambda). Vertical dotted lines indicate optimal lambda values based on minimum and 1-standard error criteria. This dimensionality reduction process identified 30 variables for subsequent inclusion in the machine-learning models to prevent overfitting.

Abbreviations: LASSO: Least absolute shrinkage and selection operator, SE: Standard error.

**Supplemental Figure 2**. Comparison of Feature Importance Across Machine Learning Models


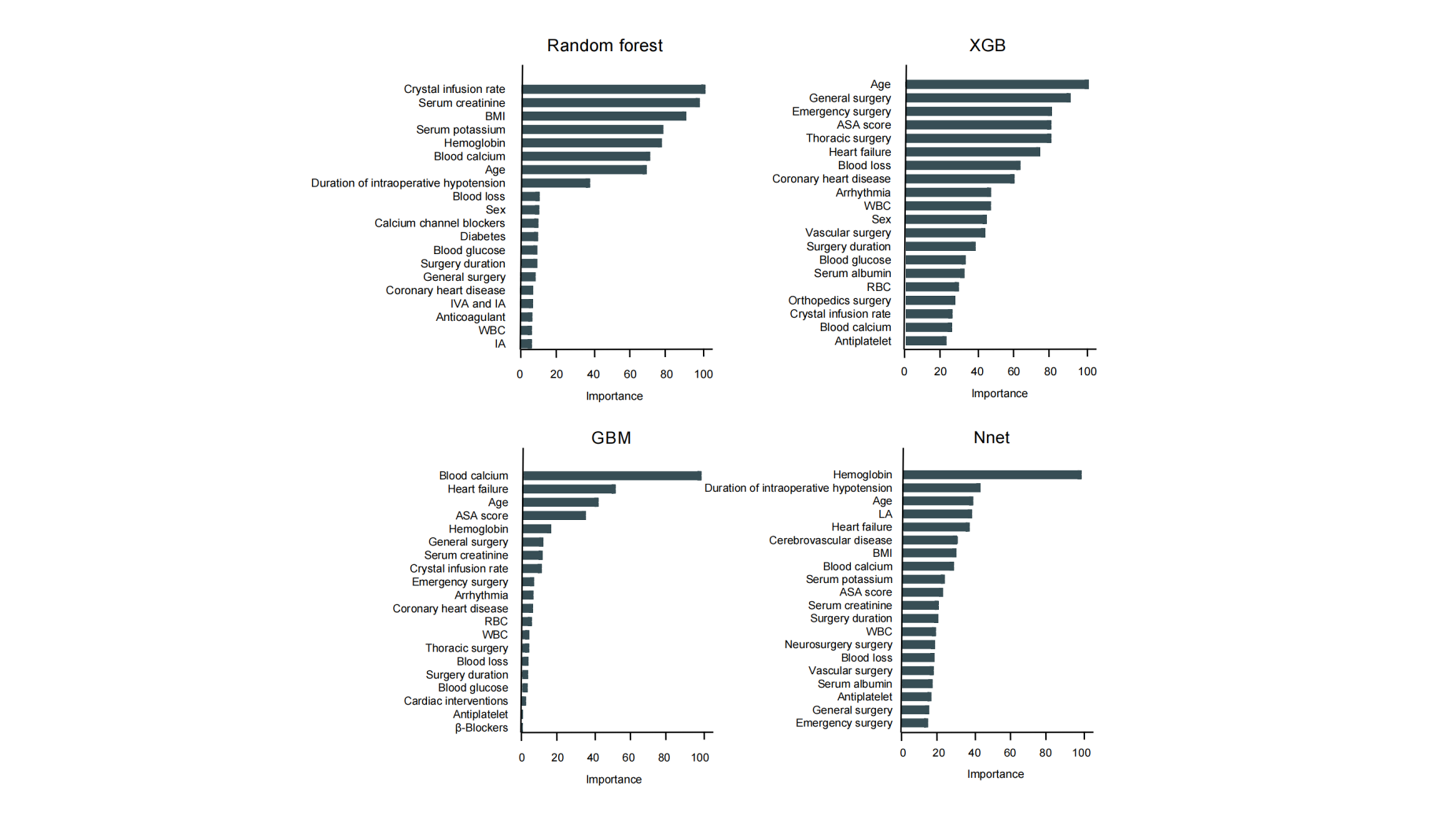


A heatmap displays the relative ranking of the top 12 selected features across the four different prediction algorithms (XGBoost, Random Forest, Logistic Regression, and SVM). Darker colors indicate higher importance rankings. The consistency of features, such as age, ASA score, and history of heart disease, across multiple models validated their robustness as key predictors of postoperative new-onset heart block.

Abbreviations: ASA: American Society of Anesthesiologists, SVM: Support vector machine, XGBoost: Extreme gradient boosting.

**Supplemental Table 1**. Detailed Hyperparameters and Selection Methods for Machine Learning Models

To ensure the reproducibility of our prediction models, we provide full configuration details for the four machine learning algorithms used in this study: Extreme Gradient Boosting (XGBoost), Neural Network (Nnet), Support Vector Machine (SVM), and Light Gradient Boosting Machine (LightGBM).

*1. Model Development Strategy*

Data Splitting: The dataset was randomly split into a training set (80%) and an internal validation set (20%).

Optimization Method: We used 10-fold cross-validation on the training set to select the optimal hyperparameters. The goal was to maximize the area under the curve (AUC) while preventing overfitting.

Feature Set: All machine learning models used the same set of 12 predictors selected via LASSO and logistic regression analyses.

*2. Hyperparameter Specifications*

| Algorithm | Hyperparameter | Value | Description & Selection Rationale |
| --- | --- | --- | --- |
| XGBoost | nrounds | 100 | Number of boosting iterations. Selected via cross-validation to ensure convergence. |
|  | eta | 0.3 | Learning rate. Controls the step size shrinkage to prevent overfitting. |
|  | max_depth | 6 | Maximum depth of a tree. Optimized to capture non-linear interactions without overfitting. |
|  | gamma | 0 | Minimum loss reduction required to make a further partition on a leaf node. |
|  | colsample_bytree | 1 | Subsample ratio of columns when constructing each tree (using all features). |
|  | min_child_weight | 1 | Minimum sum of instance weight (hessian) needed in a child. |
|  | subsample | 1 | Subsample ratio of the training instances (using all data). |
|  | objective | “reg:linear”* | The learning objective function. |
| **Neural Network** | size | 1 | Number of units in the hidden layer. Selected to maintain model simplicity and avoid overfitting. |
| (Nnet) | decay | 0 | Weight decay parameter. |
|  | maxit | 100 | Maximum number of iterations allowed. |
| **SVM** | kernel | “radial” | Radial Basis Function (RBF) kernel used to handle non-linear relationships. |
|  | gamma | 1/ncol(x) | Kernel coefficient. Default value based on feature dimensions (1/number of features). |
|  | cost | 1 | Cost of constraints violation. Optimized via grid search. |
|  | degree | 3 | Degree of the polynomial kernel (standard default). |
|  | coef0 | 0 | Independent term in kernel function. |
| **LightGBM** | num_leaves | 31 | Maximum number of leaves in one tree. Controlled to manage model complexity. |
|  | learning_rate | 0.1 | Shrinkage rate. Optimized in conjunction with nrounds. |
|  | nrounds | 100 | Number of boosting iterations. |
|  | min_data_in_leaf | 20 | Minimal number of data in one leaf. Used to prevent overfitting. |
|  | bagging_fraction | 1.0 | Fraction of data to be used for each iteration. |
|  | feature_fraction | 1.0 | Fraction of features to be used for each iteration. |

*Note: While “reg:linear” was the objective parameter setting used in the code configuration, the final model output was interpreted for binary classification probability in the ROC analysis.
